# Phytochemical Constituents of *Citrus hystrix* DC. Leaves Attenuate Inflammation via NF-κB Signaling and NLRP3 Inflammasome Activity in Macrophages

**DOI:** 10.3390/biom11010105

**Published:** 2021-01-14

**Authors:** Watunyoo Buakaew, Rungnapa Pankla Sranujit, Chanai Noysang, Yordhathai Thongsri, Pachuen Potup, Nitra Nuengchamnong, Nungruthai Suphrom, Kanchana Usuwanthim

**Affiliations:** 1 Cellular and Molecular Immunology Research Unit, Faculty of Allied Health Sciences, Naresuan University, Phitsanulok 65000, Thailand; watunyoob60@nu.ac.th (W.B.); yordhathait@nu.ac.th (Y.T.); Pachuenp@nu.ac.th (P.P.); 2 Thai Traditional Medicine College, Rajamangala University of Technology Thanyaburi, Pathum Thani 12130, Thailand; rungnapa_s@rmutt.ac.th (R.P.S.); chanai_n@rmutt.ac.th (C.N.); 3 Science Laboratory Centre, Faculty of Science, Naresuan University, Phitsanulok 65000, Thailand; Nitran@nu.ac.th; 4 Department of Chemistry, Faculty of Science and Center of Excellence for Innovation in Chemistry, Naresuan University, Phitsanulok 65000, Thailand; nungruthais@nu.ac.th

**Keywords:** *Citrus hystrix*, NLRP3, NF-κB, lupeol, inflammasome, macrophage

## Abstract

*Citrus hystrix* DC. (CH) is found in many countries in Southeast Asia. This plant has been reported for anti-microbial, anti-cancer and anti-inflammatory bioactivities. However, the anti-inflammatory and anti-inflammasome properties of the leaves remain poorly understood. This study aimed to investigate the effect of CH leaves on NLRP3 and NF-κB signaling pathways. CH leaves were sequentially extracted using hexane, ethyl acetate and 95% ethanol to give three crude extracts. An active compound, lupeol was fractionated from the ethanolic extract using chromatographic techniques, and its structure was identified and confirmed by spectroscopic methods. Anti-inflammatory activities were observed on both lipopolysaccharide-stimulated and NLRP3 adenosine triphosphate-induced macrophages. The release of pro-inflammatory cytokines (IL-1β, IL-6 and TNF-α) was analyzed by Enzyme-Linked Immunosorbent Assay (ELISA). Real-time qRT-polymerase chain reaction (PCR) was used to measure inflammatory-associated gene expression. NF-κB protein expressions were investigated using the immunoblotting technique. The active fraction of ethanolic CH leaves and lupeol significantly reduced the release of pro-inflammatory cytokines and suppressed the expression of both inflammasome genes and NF-κB proteins. The ethanolic extract of CH leaves and lupeol showed potent anti-inflammatory activities by targeting NF-κB and NLRP3 signaling pathways.

## 1. Introduction

Inflammation is the process of body response to infection or tissue injury. Innate immune cells such as natural killer (NK) cells, mast cells, neutrophils, and macrophages can recognize the existence of inflammatory inducers derived from pathogens or foreign molecules called pathogen-associated molecular patterns (PAMPs) [1]. In a tissue injury environment, damaged or dying cells release various intracellular molecules such as high-mobility group box 1 (HMGB1), heat shock proteins (HSPs) and adenosine triphosphate (ATP). These danger molecules belong to another group of inflammatory inducers called damage-associated molecular patterns (DAMPs) that can induce inflammatory responses in a non-infectious environment [2]. Among responses of immune cells to inducers, macrophages play an important role in inflammatory progression by releasing pro-inflammatory cytokines such as tumor necrosis factor-α (TNF-α) and interleukin-1β (IL-1β), and large amounts of nitric oxide (NO) to up-regulate the expression of cyclooxygenase enzyme [3]. Moreover, macrophages are pivotal cells involved in the repairing and resolution phase in an inflammatory or tissue damaged environment [4]. Failure of resolution or persistent tissue injury leads to chronic inflammation and eventually results in fibrosis and tissue dysfunction. Therefore, controlling activated macrophages by targeting and inhibiting pro-inflammatory mediators and cytokines would be a proactive method to treat inflammatory diseases.

Inflammation in macrophages can be activated via various signaling pathways including nuclear factor-κB (NF-κB) that represents a family of inducible transcription factors. Protein members in the NF-κB family are composed of NF-κB1 (p50), NF-κB2 (p52), RelA (p65), RelB and c-Rel [5]. Normally, these proteins are sequestered in cytoplasm by inhibitory protein families including IκBα. Inflammatory stimuli such as lipopolysaccharide (LPS) can stimulate signaling transduction through sensing by pattern-recognition receptors (PRRs). This event leads to degradation of IκBα, resulting in the NF-κB transcription factor translocating into the nucleus and binding to specific DNA elements to mediate pro-inflammatory gene expression. Pro-inflammatory mediator genes regulated by NF-κB include inducible nitric oxide synthase (iNOS) and cyclooxygenase-2 (COX-2) as well as various pro-inflammatory cytokines and chemokines [6]. Together with the well-recognized inflammatory signaling pathway NF-κB, inflammasome is an inflammatory mechanism that can respond to diverse sets of stimuli. To date, the activation of inflammasomes is known to depend on five protein receptors including the nucleotide binding oligomerization domain (NOD), leucine-rich repeat (LRR)-containing protein (NLR) family members including NLRP1, NLRP3 and NLRC4, as well as the proteins absent in melanoma 2 (AIM2) and pyrin [7]. NLRP3 can be activated by both PAMPs and DAMPs such as pore-forming toxin from bacteria and extracellular ATP, respectively. Signal transduction via NLRP3 results in the oligomerization of inflammasome adaptor protein named apoptosis-associated speck-like protein containing a CARD (ASC) that can recruit caspase-1 and mediate proteolytic cleavage and release of pro-inflammatory cytokine interleukine-1 beta (IL-1β) and -18 (IL-18) [8]. Therefore, NLRP3 inflammasome is a crucial target for combating inflammation.

For centuries, plants have been used as sources for therapeutic agents in various illnesses, including inflammation [9]. *Citrus hystrix* DC. (CH), also known as kaffir lime, has long been used as a medicinal herb in Thai folk medicine [10]. This tropical plant is a member of the family Rutaceae and can be found in South East Asia, particularly in Thailand, where it is a main ingredient of traditional food. This plant is 3–6 m high, the leaves part are unique with broadly ovate to ovate-oblong, dark green and fragrant [11]. CH has been reported to induce various cardioprotective and hepatoprotective bioactivities [12] as well as anti-bacterial [13] and antifungal activity [14]. A previous study [15] revealed that furanocoumarins isolated from CH peel exhibited anti-inflammatory activity by inhibiting the pro-inflammatory mediator genes NO and COX-2. However, the molecular mechanism underlying these anti-inflammatory events is unclear, while investigation of how the chemical components of CH leaves affect the inflammasome signaling pathway remains poorly understood. Here, we investigated the effect of the ethanolic extract of CH leaves and identified the active compounds that exhibited anti-inflammatory activity on LPS-induced inflammation in human monocyte-derived macrophages (MDMs) and NLRP3 inflammasome signaling pathway of ATP-induced NLRP3 inflammasomes using human myelomonocytic cell line THP-1 derived macrophages.

## 2. Materials and Methods 

### 2.1. Chemicals and Reagents

Cell culture media for in vitro culture, RPMI-1640, Antibiotic-Antimycotic and fetal bovine serum (FBS) were purchased from Gibco (Life Technologies, Grand Island, NY, USA). Phorbol-12-myristate-13-acetate (PMA), lipopolysaccharide (LPS) and adenosine triphosphate (ATP) were purchased from Sigma-Aldrich (St. Louis, MO, USA). TNF-α, IL-6 and IL-1β Enzyme-Linked Immunosorbent Assay (ELISA) kits were purchased from Sino Biological (Wayne, PA, USA). 3-(4,5-Dimethylthiazol-2-yl)-2,5-diphenyl tetrazolium bromide (MTT) and 3,3′,5,5′-tetramethylbenzidine (TMB) substrate solution and goat anti-mouse IgG (H+L) secondary antibody, HRP (cat no. 31430) were obtained from Thermo Fisher Scientific (Waltham, MA, USA). RiboZol RNA extraction reagent and dimethyl sulfoxide (DMSO) were purchased from VWR International (West Chester, PA, USA). Tetro cDNA synthesis kit and SensiFAST SYBR No-ROX kit were obtained from Bioline (Meridian Life Science, Inc., Memphis, TN, USA). RIPA (Radio-immunoprecipitation assay) buffer was purchased from Bio Basic Inc. (Amherst, NY, USA). Mouse monoclonal antibodies against human NF-κB p65 (cat. no. sc-8008), COX-2 (cat. no. sc-166475 and β-actin (cat. no. sc-47778) were obtained from Santa Cruz Biotechnology (Santa Cruz, CA, USA). Mouse monoclonal antibody against human phospho IκB-α (Ser32/26 cat. no. 9246) was purchased from Cell Signaling Technology (London, UK).

### 2.2. Plant Material Preparation and Extraction of CH Leaves

Powdered CH leaves (COA Lot.No.250818) were obtained from Khaolaor Company (Samut Prakan, Thailand). The powdered leaves (1000 g) were sequentially macerated in hexane (3000 mL) for 3 days followed by 95% ethyl acetate (3000 mL) for 3 days, 95% ethanol (3000 mL) for 3 days at room temperature and filtrated. The filtrate was then collected and evaporated by a rotary evaporator at 35 °C. The maceration procedure for each solvent was carried out in duplicate. Three crude extracts were obtained and percentage yields (% w/w dried powder) were calculated as 21.03 g (2.10% yield) of crude hexane, 39.12 g (3.91% yield) of crude ethyl acetate and 100.56 g (10.056% yield) of crude ethanolic extracts.

### 2.3. Isolation and Identification of Active Compounds

The ethanolic extract showed the highest anti-inflammatory activity and this was then fractionated by chromatographic techniques. Ten grams of crude ethanolic extract were fractionated using silica gel column chromatography. A gradient sequentially formed of hexane (100%), hexane-dichloromethane (50–50%), dichloromethane (100%), dichloromethane-methanol (90–10%), dichloromethane-methanol (50–50%) and 100% methanol was used as the mobile phase for elution. Six fractions (F1–F6) were obtained and screened for in vitro anti-inflammation assay. Fraction no. 4 (727 mg) was further fractionated using silica gel column chromatography and eluted with a gradient sequentially formed of dichloromethane-methanol to give eight subfractions (F4.1–F4.8). Ten milligrams of subfraction no. 4.6 were then fractionated using C-18 column chromatography with deionized water: acetonitrile (5:95 *v*/*v*) as the mobile phase. Finally, a white solid (1.2 mg) was obtained from this process. The structure of the isolated compound was then identified using ^1^H- and ^13^C- nuclear magnetic resonance (NMR) and Fourier-transform infrared spectrophotometer (FT-IR) mass spectroscopy techniques. NMR spectra were recorded on a Bruker AV400 (Bruker, Billerica, MA, USA) spectrometer at 400 MHz for proton and 100 MHz for carbon. A PerkinElmer Spectrum GX (PerkinElmer, Waltham, MA, USA) was used for FT-IR analysis. The mass was analyzed using gas chromatography-mass spectrometry (GC-MS) (Agilent, Santa Clara, CA, USA).

Lupeol; white solid, C_30_H_50_O, EI-MS *m/z* 426 [M]^+^, FT-IR (ATR) ν_max_ (cm^−1^): 3315, 2942, 2871, 2853, 1638, 1451, 1188, 1035. ^1^H NMR (400 MHz, CDCl_3_, δ ppm): 4.69 (*d*, *J* = 2.5 Hz H-29a), 4.56 (*dq*, *J* = 2.8, 1.4 Hz H-29b), 3.19 (*dd*, *J* = 10.7, 5.0 Hz, H-3), 2.38 (*m*, H-19), 1.91 (*m*, H-21), 1.53, 1.68 (*overlap*, H-2), 1.68 (*s*, H-30), 1.65 (*overlap*, H-13), 1.39 (*overlap*, H-7), 1.37, 1.38 (*overlap* H-18), 1.36, 1.53 (*overlap*, H-6), 1.36, 1.45 (*overlap*, H-16), 1.25 (*overlap*, H-9), 1.20, 1.40 (*overlap*, H-11), 1.17, 1.38 (*overlap*, H-22), 1.08, 1.62 (*overlap*, H-12), 1.05, 1.60 (*overlap*, H-15), 1.03 (*s*, H-26), 0.94, 1.65 (*overlap*, H-1), 0.96 (*s*, H-27), 0.94 (*s*, H-23), 0.83 (*s*, H-25), 0.79 (*s*, H-28), 0.76 (*s*, H-24), 0.67(*overlap*, H-5). ^13^C NMR (100 MHz, CDCl_3_, δ ppm): 151.1 (C-20), 109.5 (C-29), 79.2 (C-3), 55.4 (C-5), 50.6 (C-9), 48.5 (C-18), 48.1 (C-17), 48.1 (C-19), 43.0 (C-14), 41.0 (C-8), 40.2 (C-22), 39.0 (C-1), 38.9 (C-4), 38.2 (C-13), 37.3 (C-10), 35.7 (C-16), 34.4 (C-7), 30.0 (C-21), 28.1 (C-23), 27.6 (C-2), 27.6 (C-15), 25.3 (C-12), 21.1 (C-11), 19.5 (C-30), 18.5 (C-28), 18.2 (C-6), 16.3 (C-25), 16.1 (C-26), 15.5 (C-24), 14.7 (C-27).

### 2.4. Primary Human Monocyte Isolation and Differentiation

Human monocyte-derived macrophages (MDMs) were isolated from buffy coat obtained from the blood bank of Naresuan University Hospital, Phitsanulok, Thailand. This project received ethical approval from the Human Ethics Committee, Naresuan University Ethics Committee (IRB no. 1065/61). Human peripheral blood mononuclear cells (PBMCs) were isolated by density gradient centrifugation using Lymphoprep ™ (Axis-Shield PoC AS, Rodeløkka, Oslo, Norway), followed by 46% Percoll (GE Healthcare Bio-Sciences AB, Uppsala, Sweden). Cells were maintained in RPMI-1640 supplemented with 10% FBS and 1% Antibiotic-Antimycotic (Thermo Fisher Scientific, Inc., Waltham, MA, USA) in a humidified atmosphere of 5% CO_2_ at 37 °C for one week with medium replacement every 2–3 days. Human MDMs from this process were confirmed for phenotype using flow cytometry analysis.

### 2.5. Differentiation of THP-1 Cells and Inflammasome Activation

THP-1 cells obtained from ATCC were maintained in RPMI-1640 supplemented with 10% FBS and 1% Antibiotic-Antimycotic (Thermo Fisher Scientific, Inc., Waltham, MA, USA) in a humidified atmosphere of 5% CO_2_ at 37 °C. Cells were differentiated to macrophages by stimulating with 100 nM phorbol-12-myristate-13-acetate (PMA) (Sigma-Aldrich, St. Louis, MO, USA) for 3 days followed by a PMA-free complete medium replacement for a further four days. The expression of macrophage phenotypic markers was analyzed using the flow cytometry technique. For NLRP3 inflammasome activation, the method was performed by inducing PMA-treated THP-1 with 1 µg/mL LPS of *Escherichia coli* O55:B5 (Sigma-Aldrich, St. Louis, MO, USA) in RPMI-1640 supplemented with 10% FBS and 1% antibiotic for 3 h. After LPS priming, the cell culture medium was replaced with RPMI-1640 containing 5 mM adenosine triphosphate (ATP) (Sigma-Aldrich, St. Louis, MO, USA) for 1 h following the method described previously [16]. To evaluate the inhibitory effect of CH extract and identify compounds on inflammasome activation, the compounds were co-treated with ATP.

### 2.6. Flow Cytometry Analysis

The expressions of macrophages cell surface markers were evaluated by staining with PE-conjugated anti-CD14 and FITC-conjugated anti-CD16 (BD Biosciences, San Jose, CA, USA). Harvested cells were centrifuged and resuspended in FACS buffer containing fluorescence-conjugated antibodies at 4 °C for 1 h. After incubation, the cells were washed three times with phosphate-buffered saline (PBS) and analyzed using an FC 500 Flow Cytometer (Beckman Coulter, Inc., Indianapolis, IN, USA). FITC-conjugated mouse IgG1, κ isotype control and PE-conjugated mouse IgG1, κ isotype control (BioLegend, Inc., San Diego, CA, USA) were used as negative control.

### 2.7. Cell Viability Measurement

Human MDMs and THP-1-derived macrophages were seeded in a 96-well plate at a density of 5 × 10^4^ cells/well and incubated for 24 h. Cells were treated with different concentrations of extract for 24 h. Cell viability was analyzed using MTT assay modified according to the protocol described elsewhere [17]. Briefly, cells were washed with 100 µL phosphate-buffered saline (PBS) and then incubated with 0.5 mg/mL of MTT (Thermo Fisher Scientific, Waltham, MA, USA) in RPMI-1640 serum-free medium in a humidified atmosphere of 5% CO_2_ at 37 °C for 3 h. Supernatants were discarded and 50 µL of 100% dimethyl sulfoxide (DMSO) (VWR International, West Chester, PA, USA) was added to solubilize the formazan crystals for 15 min. The absorbance was measured at 570 nm using a microplate reader. Cell viability percentage was calculated and the concentration that maintained cell viability greater than or equal to 90% was selected for further experiment.

### 2.8. Evaluation of Anti-Inflammatory Effect

MDMs and THP-1-derived macrophages were seeded in a 24-well plate at a density of 2 × 10^5^ cells/well in RPMI-1640 supplemented with 10% FBS and 1% antibiotic for 24 h. The cells were pre-treated with extract for 1 h, then co-treated with 100 ng/mL LPS of *Escherichia coli* O55:B5 (Sigma-Aldrich, St. Louis, MO, USA) and maintained at 37 °C in cell culture condition for a further 12 h. Positive control condition was cells co-treated with the anti-inflammatory drug dexamethasone (0.1 nM).

### 2.9. Enzyme-Linked Immunosorbent Assay (ELISA)

The cell culture supernatant was collected and evaluated for expression of IL-1β, IL-6 and TNF-α pro-inflammatory cytokines using sandwich ELISA assay according to the manufacturer’s protocol (Sino Biological, Wayne, PA, USA). Absorbance of the reaction was measured at 450 nm using an EnSpire^®^ Multimode microplate reader (PerkinElmer, Inc., Waltham, MA, USA).

### 2.10. Real-Time Quantitative RT-PCR

Cells from each experimental condition were extracted for RNA using RiboZol RNA extraction reagent (VWR International, West Chester, PA, USA) following the manufacturer’s protocol. Total RNA was converted to complementary DNA (cDNA) by polymerase chain reaction (PCR) using a Tetro cDNA synthesis kit (Bioline, London, UK). Gene expression levels were evaluated by qPCR using SensiFAST™ SYBR No-ROX kit (Bioline, London, UK). The reaction was performed in a CFX96 Touch Real-Time PCR Detection System (Bio-Rad Laboratories, Inc., Hercules, CA, USA). The PCR step was composed of polymerase activation at 95 °C for 1 min followed by 45 cycles of denaturation step at 95 °C for 15 s and then annealing and extension at 60 °C for 1 min. Human beta actin (ACTB) gene was used as the housekeeping gene. All data were analyzed by normalized gene expression using a 2^−ΔΔCT^ method [18]. All primer sequences are shown in Table 1.

### 2.11. SDS-PAGE and Western Blot Analysis

The cells in each condition were lysed using ice-cold RIPA buffer (Bio Basic Inc., Amherst, NY, USA) with protease and phosphatase inhibitor cocktails (Thermo Fisher Scientific, Waltham, MA, USA) for 30 min, and then centrifuged at 12,000 rpm for 30 min at 4 °C. The supernatants were collected, and protein concentration was determined using Bradford reagent. Equal amounts of proteins were loaded in 12% SDS-polyacrylamide gel electrophoresis (PAGE) and then transferred to a 0.2 µm polyvinylidene fluoride membrane (Bio-Rad Laboratories, Inc., Hercules, CA, USA). The membrane was blocked at 4 °C overnight with 5% bovine serum albumin (Capricorn Scientific GmbH, Hesse, Germany) in Tris-buffered saline with Tween 20 (TBST) buffer. Primary antibodies against phospho IκB-α (Cell Signaling Technology, London, UK), NF-κB P65, and COX-2 (Santa Cruz, CA, USA) were probed onto the membrane at room temperature for 1 h. Then, the membrane was washed with TBST buffer and incubated with horseradish peroxidase-conjugated goat anti-mouse IgG (H + L) secondary antibody (Thermo Fisher Scientific, Waltham, MA, USA) at room temperature for 1 h. Protein bands were observed using chemiluminescence substrate for 5 min and visualized in a ChemiDoc XRS+ Imaging System (Bio-Rad Laboratories, Inc., Hercules, CA, USA). Protein intensity was measured using Image Studio Lite software (LI-COR Corporate, Lincoln, NE, USA).

### 2.12. Statistical Analysis

GraphPad Prism Software version 6 (GraphPad Software Inc., San Diego, CA, USA) was used to analyze the data. Three-independent experiments were performed in triplicate. All data were expressed as mean ± standard deviation. One-way ANOVA followed by Tukey’s multiple comparison post-hoc test were used to compare the means. A value of *p* < 0.05 was considered statistically significant.

## 3. Results

### 3.1. Extraction and Fractionation

**Figure 1:**
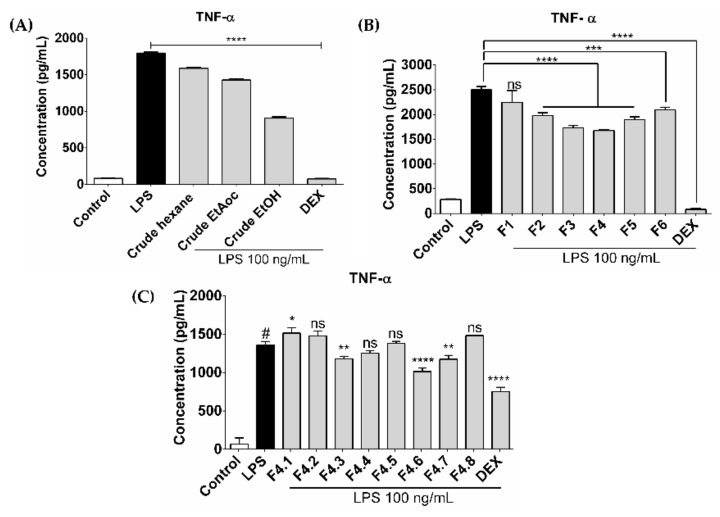
Anti-inflammatory effect of crude, fractions, and sub-fractions *C. hystrix* leaves on TNF-α production. Level of TNF-α release after treatment with crude extracts (**A**), fractions (**B**) and sub-fractions (**C**) in an LPS-induced human MDMs model. Data are presented as mean ± SD. * *p* < 0.05; ** *p* < 0.01; *** *p* < 0.001; **** *p* < 0.0001 compared to LPS treatment. LPS: Lipopolysaccharide-stimulated macrophages; Dex: Dexamethasone; F: Fraction.

A total of 1000 g of dried CH powder was processed into three types as 21.03 g (2.10% yield) of crude hexane, 39.12 g (3.91% yield) of crude ethyl acetate and 100.56 g (10.056% yield) of crude ethanolic extracts. The extracts were tested for anti-inflammatory activities to compare against the other solvents. Results showed that the crude ethanolic extract gave the highest activity (Figure 1A), and this was then fractionated by chromatographic techniques. Six fractions (F1–F6) were obtained and screened for in vitro anti-inflammation assay. Fraction no. 4 (727 mg) showed the most activity (Figure 1B) and this was further fractionated using silica gel column chromatography and eluted with a gradient sequentially formed of dichloromethane-methanol to give eight subfractions (F4.1–F4.8). Subfraction no. 4.6 showed the highest inhibitory activity (Figure 1C) and was labeled as the active fraction (CHAF).

### 3.2. Identification of an Active Compound Isolated from CHAF

**Figure 2:**
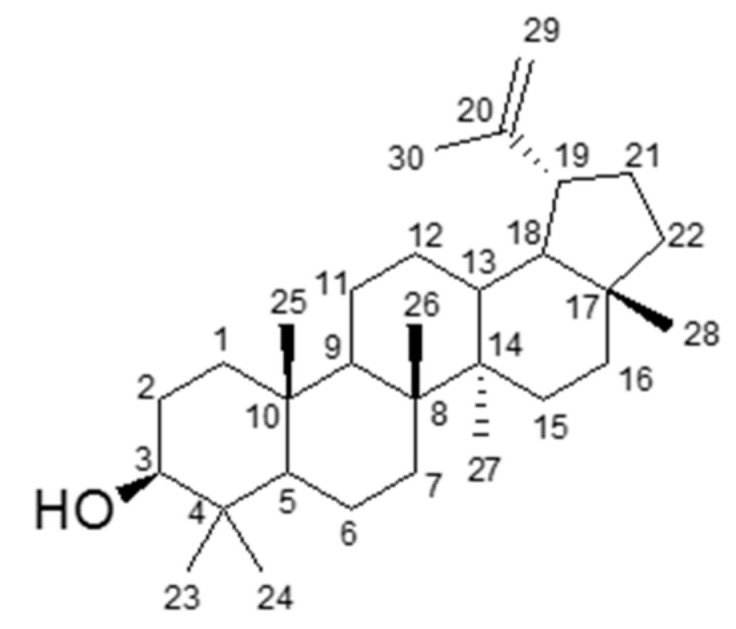
Chemical structure of lupeol.

Chromatographic fractionation of the ethanolic crude extract and its subfraction no. 4.6 (CHAF) resulted in the isolation of an active compound. The structure was elucidated on the basis of spectroscopic data (Figures S1–S4). Moreover, identification of this compound was also performed by computer matching its recorded mass spectrum with a standard library, Wiley7n, at 95% matching. The obtained spectra were compared with reported data [19,20,21] and the structure was identified as a known pentacyclic triterpenoid, namely lupeol (Figure 2).

### 3.3. Cell Surface Marker Analysis of Human MDMs by Flow Cytometry

**Figure 3:**
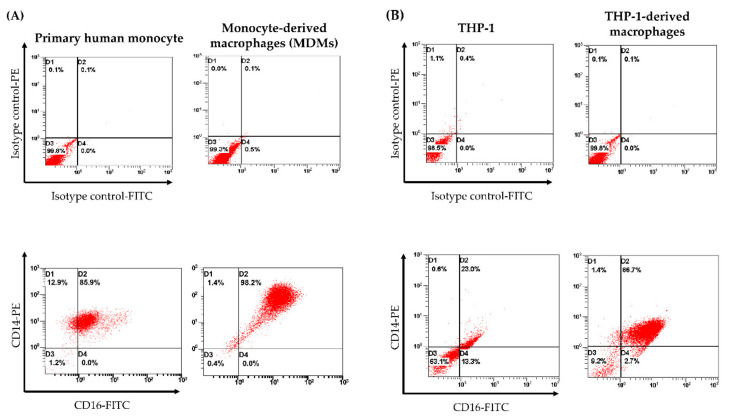
Flow cytometry analysis of CD14 and CD16 expression. (**A**) Expression profile of surface markers in primary human monocytes and MDMs obtained from buffy coat of a healthy donor. Percentages of positive CD14/CD16 in monocytes and MDMs were 85.9% and 98.7%, respectively. (**B**) Expression of cell surface markers in THP-1, CD14/CD16 expression was 23.0%. After differentiation with PMA, CD14/CD16 expression on cell surface increased to 86.7%.

To determine the differentiated phenotypes of human MDMs and THP-1-derived macrophages, CD14-PE and CD16-FITC-conjugated antibodies were analyzed for expression of cell surface markers. Expressions of CD14/CD16 double positive in primary human monocytes and THP-1 were 85.9% and 23.0%, respectively. By contrast, 7-day macrophage differentiation in human monocytes and THP-1 yielded the up-regulation of CD14/CD16 on cell surfaces that were 98.2% and 86.7%, respectively (Figure 3). From this experiment, MDMs and THP-1-derived macrophages shared similar macrophage phenotypes based on CD14/CD16 expression.

### 3.4. CHAF and Lupeol Suppression on Production of Pro-Inflammatory Mediators in LPS-Stimulated Human MDMs

**Figure 4:**
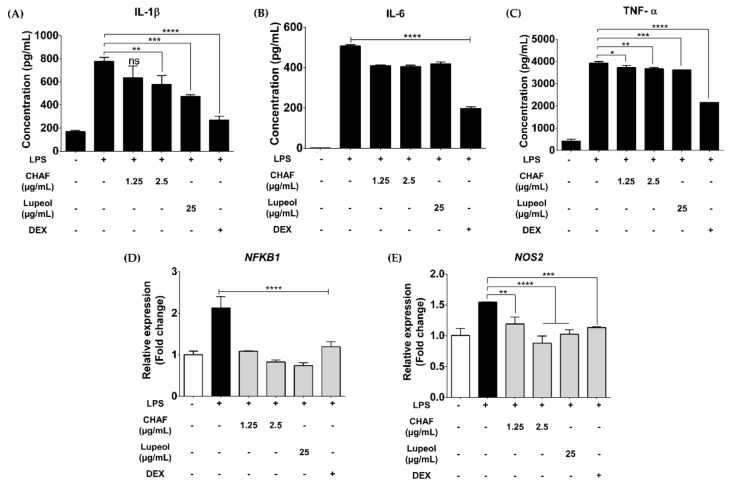
Anti-inflammatory effects of CHAF and lupeol in LPS-stimulated human MDMs. MDMs were pre-treated with CHAF and lupeol for 1 h, followed by treatment with LPS (100 ng/mL) for 12 h. Level of pro-inflammatory cytokines IL-1β (**A**), IL-6 (**B**) and TNF-α (**C**). CHAF and lupeol were also affected by pro-inflammatory mRNA expression including *NFKB1* (**D**), and *NOS2* (**E**). Data are presented as mean ± SD. * *p* < 0.05; ** *p* < 0.01; *** *p* < 0.001; **** *p* < 0.0001 compared to LPS treatment. LPS: Lipopolysaccharide-stimulated macrophages; Dex: Dexamethasone; CHAF: subfraction no. 4.6.

Cellular cytotoxicity concentrations of CHAF on MDMs and THP-1-derived macrophages were performed (Figure S5). The anti-inflammatory effects of CHAF and lupeol were observed in LPS-stimulated MDMs. Pre-treatment with CHAF and lupeol significantly reduced the production of pro-inflammatory cytokines IL-1β (Figure 4A), IL-6 (Figure 4B) and TNF-α (Figure 4C) compared to LPS-stimulated condition alone. Moreover, the mRNA expression of pro-inflammatory mediators including *NFKB1* (Figure 4D) and *NOS2* (Figure 4E) were suppressed under the CHAF and lupeol pre-treatment conditions.

### 3.5. Effect of CHAF and Lupeol on NLRP3 Inflammasome mRNA Expression in THP-1

**Figure 5:**
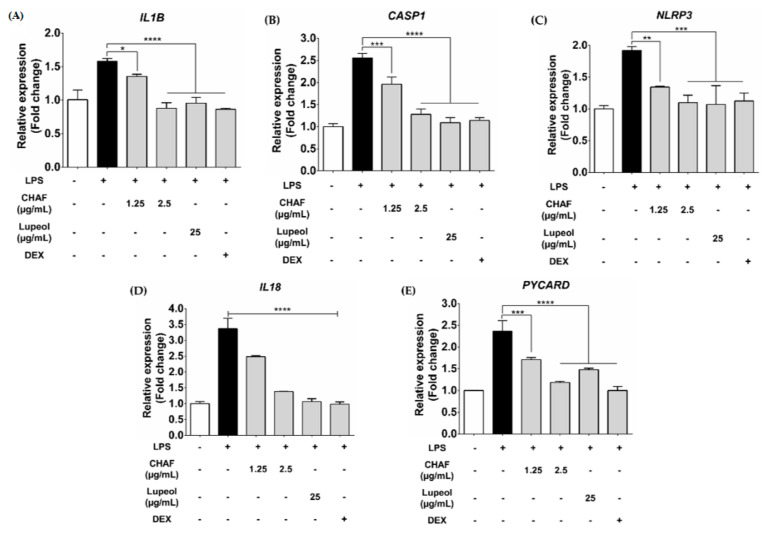
NLRP3 inflammasome mRNA expression was suppressed by CHAF and lupeol. THP-1-derived macrophages were primed with LPS (1 µg/mL) for 3 h, followed by ATP (5 mM) with or without CHAF and lupeol for 1 h. Effect of CHAF and lupeol on the expression of NLRP3 inflammasome-associated genes including *IL1B* (**A**), *CASP1* (**B**), *NLRP3* (**C**), *IL18* (**D**) and *PYCARD* (**E**). Data are presented as mean ± SD. * *p* < 0.05; ** *p* < 0.01; *** *p* < 0.001; **** *p* < 0.0001 compared to the combination of LPS and ATP treatments. LPS: Lipopolysaccharide-stimulated macrophages; Dex: Dexamethasone; CHAF: subfraction no. 4.6.

This experiment focused on investigating the anti-inflammasome effect of CHAF and lupeol. LPS priming and ATP treatment in THP-1-derived macrophages significantly increased the expression of canonical inflammasome-associated genes including *IL1B* (Figure 5A), *CASP1* (Figure 5B), *NLRP3* (Figure 5C)*, IL18* (Figure 5D) and *PYCARD* (Figure 5E). By contrast, ATP co-treated with the indicated concentration of CHAF and lupeol significantly suppressed the expression of mRNA level compared to non-treatment with extract groups.

### 3.6. The Effect of CHAF and Lupeol on NF-κB Signaling and COX-2 Proteins

**Figure 6:**
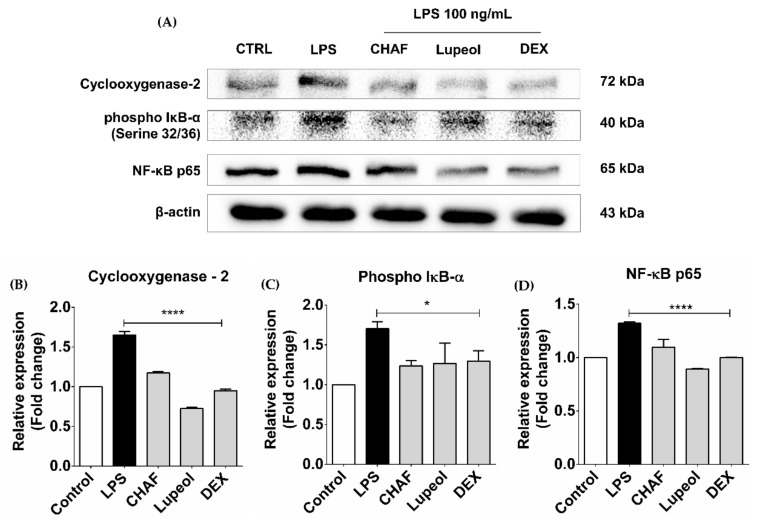
Expression of COX-2, phospho IκB-α and NF-κB p65 proteins in LPS-stimulated THP-1-derived macrophages. THP-1 cells were differentiated to macrophages using PMA, then the cells were pre-treated with CHAF (2.5 µg/mL) and lupeol (25 µg/mL) for 30 min, followed by treatment with LPS (100 ng/mL) for 1 h. Specific antibodies against phospho IκB-α, NF-κB p65 and COX-2 (**A**) were used to detect protein expression. Beta actin was used as the internal control. A densitometry scanner was used to quantify the level of protein normalized to internal control and the relative expression of phospho IκB-α (**B**), NF-κB p65 (**C**) and COX-2 (**D**) are shown. Data are presented as mean ± SD. * *p* < 0.05; ** *p* < 0.01; *** *p* < 0.001; **** *p* < 0.0001 compared to LPS treatment. LPS: Lipopolysaccharide-stimulated macrophages; Dex: Dexamethasone; CHAF: subfraction no. 4.6.

To further investigate the anti-inflammatory activity of CHAF and lupeol, the classical NF-κB protein expressions in THP-1-derived macrophages were measured as well as the expression of pro-inflammatory enzyme COX-2. In this experiment, IκB-α phosphorylated form and NF-κB p65 protein expressions were used to confirm the effect on NF-κB signaling pathway. Results showed that LPS-induced the expression of phospho-IκBα and NF-κB p65 transcription factors (Figure 6). However, pre-treatment with CHAF and lupeol decreased the expression of both proteins. In addition, the level of COX-2 protein expression was down-regulated by CHAF and lupeol treatment compared to LPS-stimulated condition.

## 4. Discussion

Some parts of *C. hystrix* have been reported to induce anti-inflammatory activity such as the peel. Nevertheless, information regarding the leaves remains limited and the effect on NLRP3 inflammasome still lacks evidence. Here, we examined the anti-inflammatory and anti-inflammasome activities of an active subfraction of the ethanolic extract of *C. hystrix* leaves (CHAF) and its active compound namely lupeol. Both CHAF and lupeol exhibited anti-inflammatory properties by suppressing the production of pro-inflammatory cytokines including IL-1β, IL-6 and TNF-α, as well as the expression of pro-inflammatory genes *NFKB1* and *NOS2.* Similar results were reported from other parts of this plant, including the essential oil that inhibited the activity of 5-lipoxygenase [22]. Coumarins [23] and furanocoumarins isolated from the peel inhibited NO production, iNOS and COX-2 gene expressions in RAW264.7 macrophages [15]. In this study, CHAF inhibited the NO signaling pathway by suppressing the expression of *NOS2* in LPS-stimulated MDMs.

LPS induces pro-inflammatory cytokine production via binding to Toll-like receptor (TLR) 4 and causes nuclear translocation of NF-κB through MyD88-dependent signaling. The release of TNF-α requires a signal from MyD88 and TRIF-dependent pathways. However, the activation of TNF-α promoter is MyD88-dependent but not TRIF [24]. In addition, we observed the ability of CHAF to inhibit both production of TNF-α and expression of *NFKB1* gene. Taken together, our result suggests that CHAF might regulates the inflammation via MyD88-dependent pathways. Another possible mechanism of action of lupeol on inhibiting inflammatory activation in this study is rely on the receptor level. The study from Ming Xu et al. showed that lupeol can down-regulate TLR4 in coxsackievirus B3-induced viral myocarditis in mice [25]. To determine the exactly target of lupeol, further investigation on molecular binding is required to confirm the effect of CHAF targeting on molecules in MyD88 signaling pathways.

Apart from NF-ĸB signaling pathway, we found the inhibitory effect of CHAF on NLRP3 inflammasome signaling pathway. Our study revealed the first evidence that CHAF has the ability to attenuate the expression of selected *NLRP3* inflammasome gene expressions in THP-1-derived macrophage response to LPS and ATP treatment. This phenomenon can be observed in lupeol treatment, especially *NLRP3* gene suppression (Figure 5C).

The effect of lupeol in NLRP3 inflammasome gene expressions on astrocytes has been reported by Markley et al. [26]. However, our study focused on the mRNA level. Confirmation of the effect of CHAF on the NLRP3 inflammasome requires further investigation in signaling protein interaction.

To identify the active compounds which play an important role in suppressing inflammation and inflammasome activation, we isolated and identified lupeol as an active compound in the extract of *C. hystrix* leaves. Lupeol, a pentacyclic lupane-type triterpene contains significant biological properties such as antiprotozoal activity [27,28], anticancer [29], antimicrobial activity [30] and anti-inflammation [31]. This triterpene is found in various types of plants such as *Bridelia scleroneura* [32], *Millettia versicolor* [33] and *Euclea natalensis* [27]. Our study showed that *C. hystrix* leaves are a lupeol source, concurring with the findings of Anuchapreeda et al. [34].

The anti-inflammatory property of lupeol has been reported from various types of plants. Geetha et al. reported that lupeol isolated from stem bark of *Crataeva nurvala* reduced *Mycobacterium tuberculosis*-induced paw edema in rat [35], while lupeol isolated from *Crataeva religiosa* suppressed the function of various immune cells including CD4^+^ T cell [36]. A study by Vasconcelos et al. also demonstrated the potent function of lupeol isolated from *Diplotropis ferruginea* Benth. (Fabaceae) that reduced lung inflammation in bronchial asthma mice model [37].

**Figure 7:**
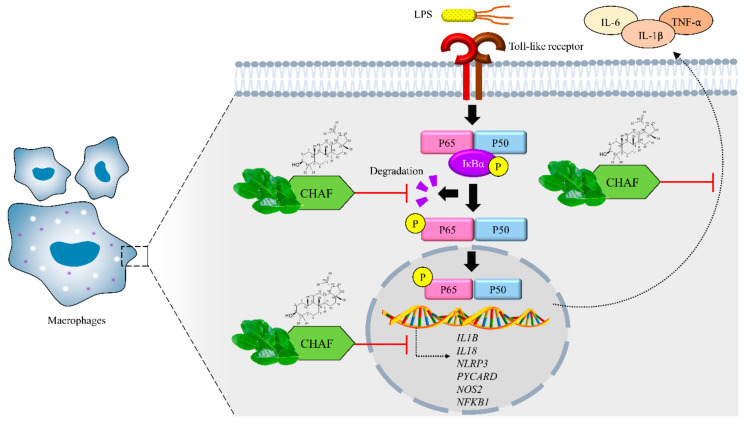
Anti-inflammatory and anti-inflammasome mechanisms of CHAF. Binding of LPS to Toll-like receptor resulting in IκB-α phosphorylation and degradation. The active form of NF-κB translocates into the nucleus and induces the transcription of pro-inflammatory and inflammasome-associated genes as well as release of cytokines such as IL-1β, IL-6 and TNF-α. CHAF attenuates the inflammation process by suppressing IκB-α phosphorylation and NF-κB p65 subunit expression. This inhibition leads to down-regulating inflammatory-associated gene expressions and decreasing the release of pro-inflammatory cytokines.

In summary, this study provides information on the anti-inflammatory and anti-NLRP3 inflammasome properties of *C. hystrix* leaves (Figure 7). Moreover, our research illustrates the source of lupeol isolated from CH leaves. Results suggested that *C. hystrix* could be used as a novel way to treat inflammatory and inflammasome-associated diseases.

## 5. Conclusions

The bioactivity of *C. hystrix* leaves exerted anti-inflammatory properties in LPS-stimulated human macrophages by reducing the production of pro-inflammatory cytokines IL-1β, IL-6, TNF-α and NF-κB signaling molecules as well as inflammatory-associated genes. The extract of *C. hystrix* leaves exhibited promising anti-inflammasome activation via down-regulation of the expression of NLRP3-associated genes including IL-1β, IL-18, caspase-1, ASC and NLRP3. Findings suggest the potential of *C. hystrix* leaves as a source of lupeol and other chemical constituents to identify novel anti-inflammation and anti-inflammasome agents.

## Figures and Tables

**Table 1:** Primer sequences used in this study.

Gene	Description	Forward (5′ → 3′)	Reverse (5′ → 3′)
*IL1B*	Interleukin 1 beta	AGCTACGAATCTCCGACCAC	CGTTATCCCATGTGTCGAAGAA
*IL18*	Interleukin 18	GAAGATGCCAGGGGTAATGA	TACCTGCCCCAAACTGAAAC
*CASP1*	Caspase-1	CTTGCTTGAAATGTGCTCCA	AGTGGCATCCCTGTTTGTTC
*NLRP3*	NLR family pyrin domain containing 3	ACAAACTCATGGTGGCTTCC	GGCCAGAAGAAAAGCAAGTG
*PYCARD*	PYD and CARD domain containing	TGACGGATGAGCAGTACCAG	AGGATGATTTGGTGGGATTG
*NFKB1*	Nuclear factor kappa B subunit 1	AACAGAGAGGATTTCGTTT	TTTGACCTGAGGGTAAGAC
*NOS2*	Nitric oxide synthase 2	TTCAGTATCACAACCTCAGCAAG	TGGACCTGCAAGTTAAAAT
*ACTB*	Actin beta	AGAAAATCTGGCACCACACC	CCATCTCTTGCTCGAAGTCC

## Supplementary Materials

The following are available online at https://www.mdpi.com/2218-273X/11/1/105/s1, Figure S1: ^1^H NMR spectrum of lupeol (at 400 MHz in CDCl_3_), Figure S2: ^13^C NMR spectrum of lupeol (at 100 MHz in CDCl_3_), Figure S3: FT-IR spectrum of lupeol, Figure S4: EI-MS spectrum of lupeol, Figure S5: Cellular cytotoxicity of CHAF and Lupeol.
