# Supplementary material for: Phytochemical Constituents of Citrus hystrix DC. Leaves Attenuate Inflammation via NF-κB Signaling and NLRP3 Inflammasome Activity in Macrophages
Source: Biomolecules. 2021 Jan 14;11(1):105. doi: 10.3390/biom11010105 (PMC7829879; doi:10.3390/biom11010105)
Supplement: Supplementary file 1 [file biomolecules-11-00105-s001.pdf]

biomolecules

Supplementary materials

Phytochemical Constituents of *Citrus hystrix* DC. Leaves Atten-Uate Inflammation via Nf-κB Signaling and Nlrp3 Inflam-Masome Activity in Macrophages

Watunyoo Buakaew [1], Rungnapa Pankla Sranujit [2], Chanai Noysang [2], Yordhathai Thongsri[1], Pachuen Potup [1], Nitra Nuengchamnong[3], Nungruthai Suphrom [4] and Kanchana Usuwanthim[1,*]

[1] Cellular and Molecular Immunology Research Unit, Faculty of Allied Health Sciences, Naresuan University, Phitsanulok 65000, Thailand
[2] Thai Traditional Medicine College, Rajamangala University of Technology Thanyaburi, Pathum Thani 12130, Thailand
[3] Department of Chemistry, Faculty of Science and Center of Excellence for Innovation in Chemistry, Naresuan University, Phitsanulok 65000, Thailand
[4] Science Laboratory Centre, Faculty of Science, Naresuan University, Phitsanulok 65000, Thailand; Nitran@nu.ac.th
* Correspondence: Kanchanau@nu.ac.th; Tel.: +66-55-966-411

Citation: Buakaew, W.; Sranujit, R.P.; Noysang, C.; Thongsri, Y.; Potup, P.; Nuengchamnong, N.; Suphrom, N.; Usuwanthim, K. Phytochemical Constituents of *Citrus hystrix* DC. Leaves Atten-Uate Inflammation via Nf-κB Signaling and Nlrp3 Inflam-Masome Activity in Macrophages. **2021**, *11*, 105. https://doi.org/10.3390/biom11010105

Received: 16 December 2020
Accepted: 12 January 2021
Published: 14 January 2021

Publisher's Note: MDPI stays neutral with regard to jurisdictional claims in published maps and institutional affiliations.

Copyright: © 2021 by the authors. Licensee MDPI, Basel, Switzerland. This article is an open access article distributed under the terms and conditions of the Creative Commons Attribution (CC BY) license (http://creativecommons.org/licenses/by/4.0/).

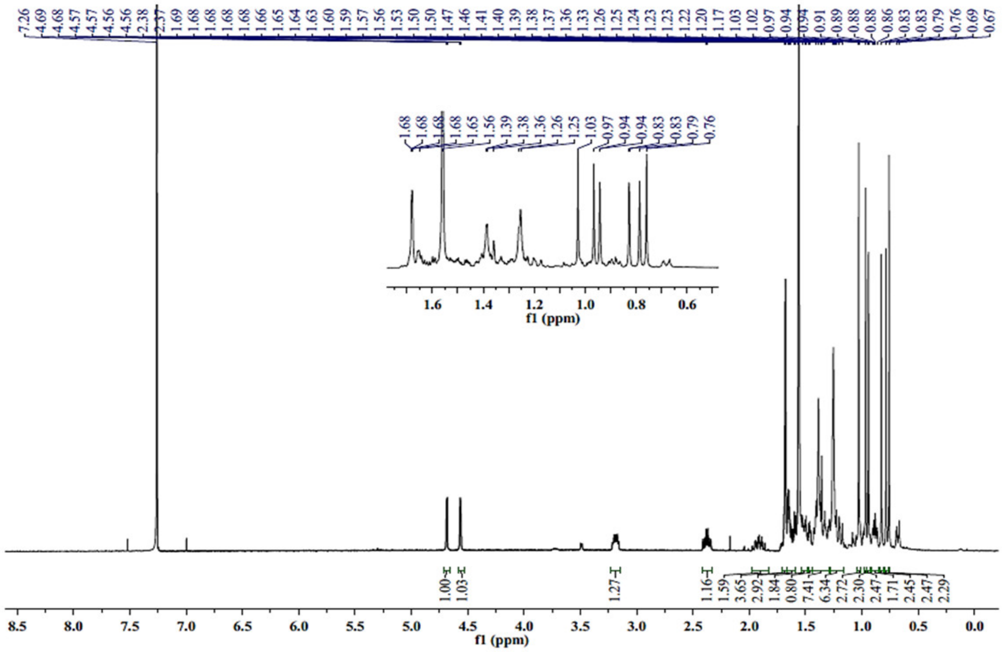

Figure S 1 ^{1}H NMR spectrum of lupeol (at 400 MHz in $CDCl_3$)

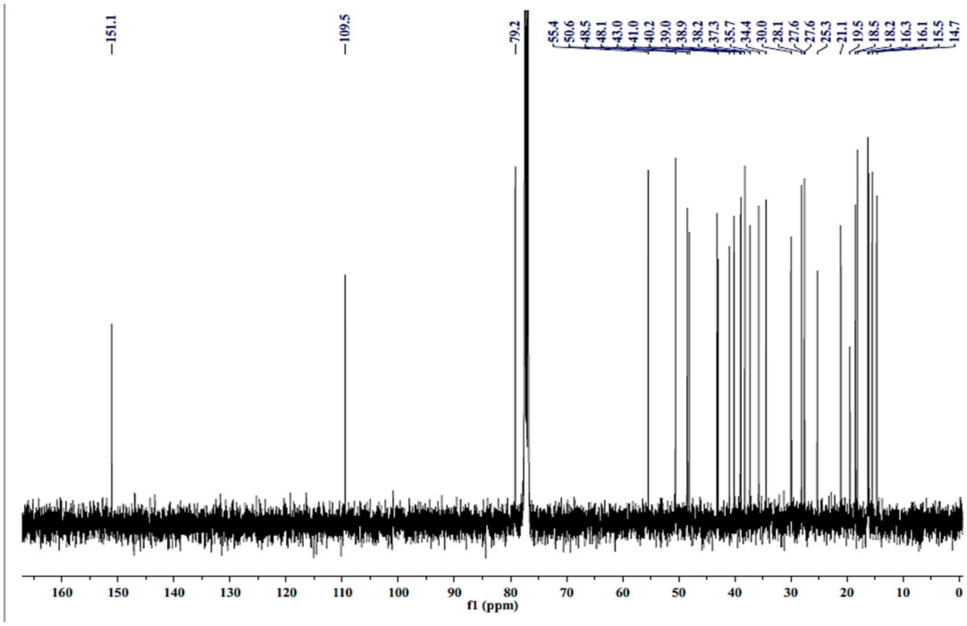

Figure S 2 NMR spectrum of lupeol (at 100 MHz in $CDCl_3$)

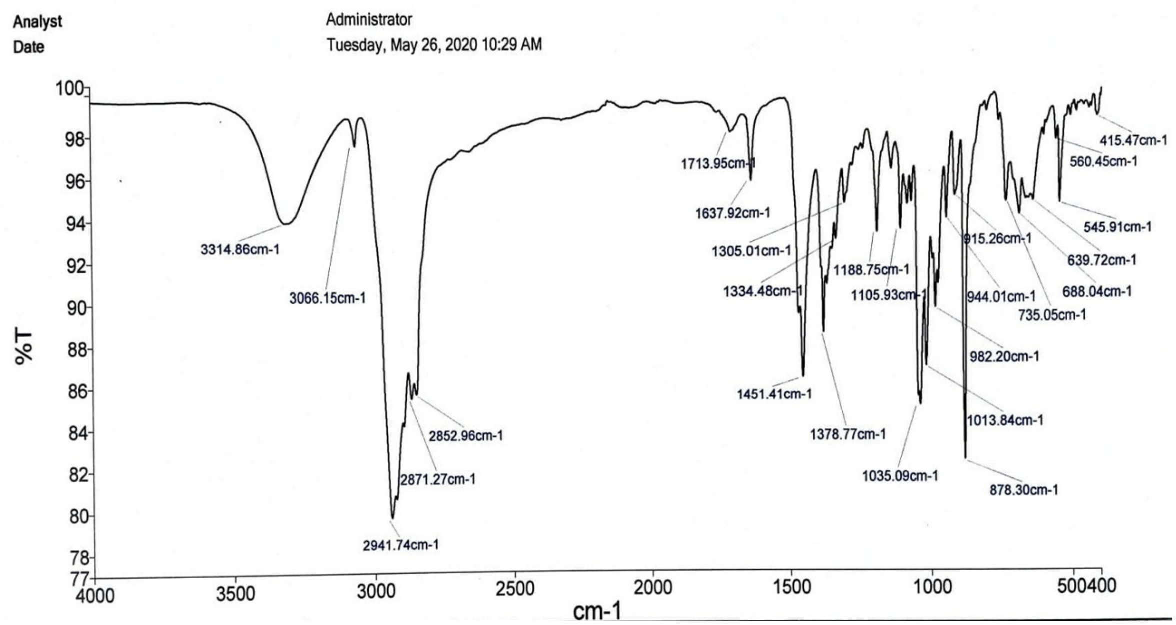

Figure S 2 FT-IR spectrum of lupeol

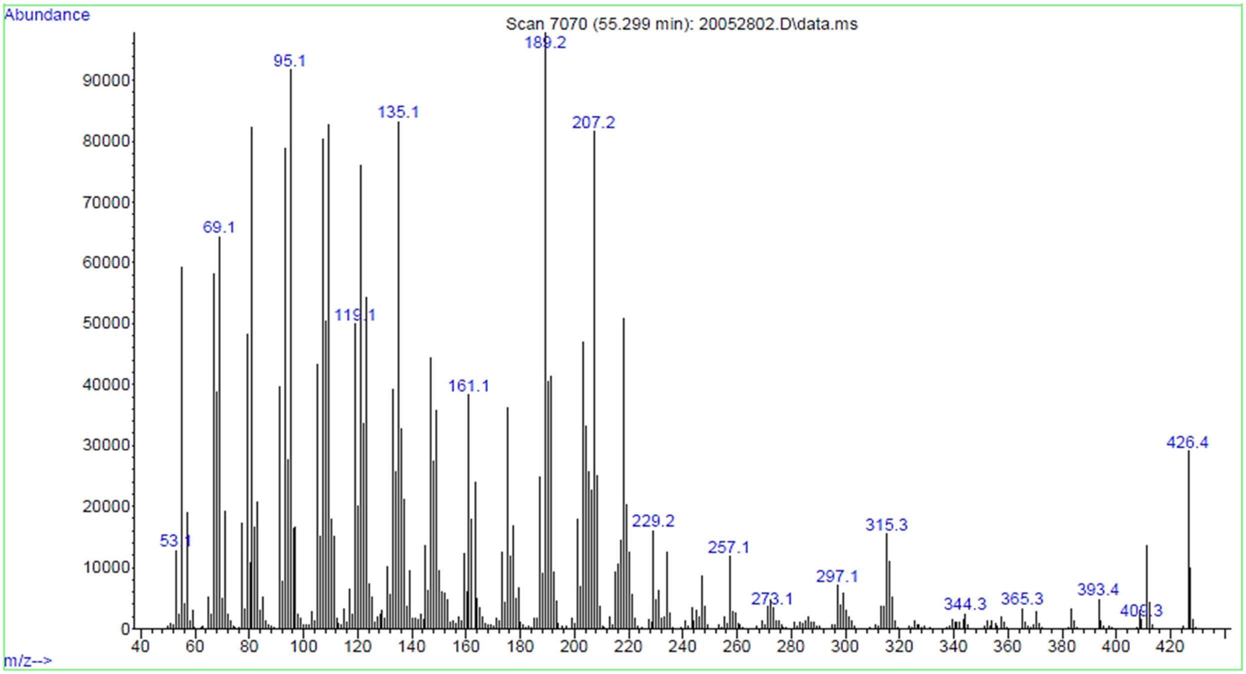

Figure S 3 EI-MS spectrum of lupeol

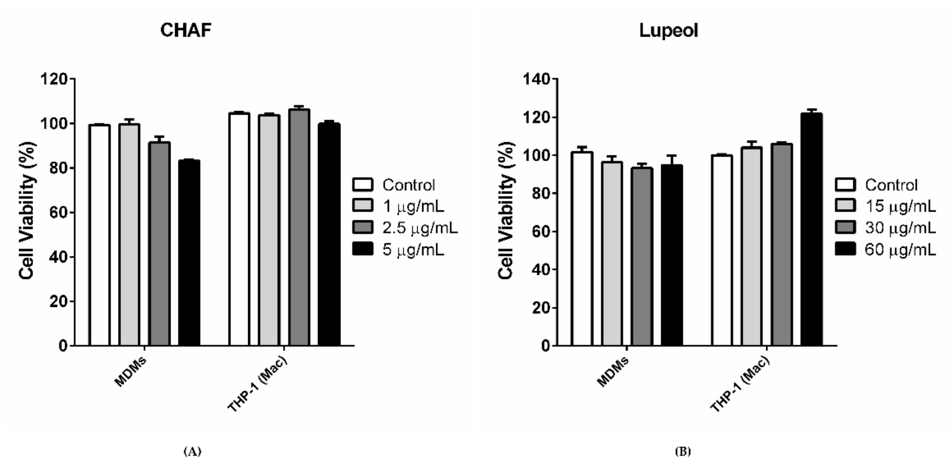

Figure S 4 Cellular cytotoxicity of CHAF and Lupeol. Cellular cytotoxicity of CHAF and Lupeol. (A) The effect of CHAF and (B) Lupeol on cell viability percentage in human MDMs and THP-1-derived macrophages. Cells were seeded in 96-well plate at density 5 × 10^4 cell/well, then treated with indicated concentration of CHAF and incubated for 24 h. The cell viability was determined using MTT assay. The final concentration of DMSO used in this study was < 0.1%. The data are presented as mean ± SD.

3. Western blotting pictures

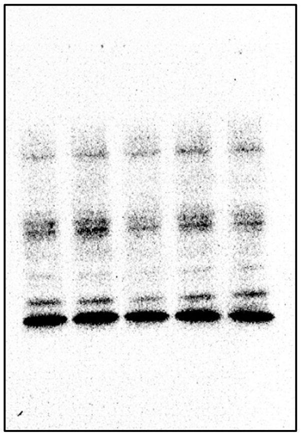

phospho IκB-α

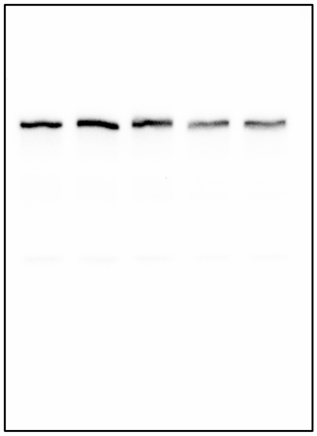

NF-κB p65

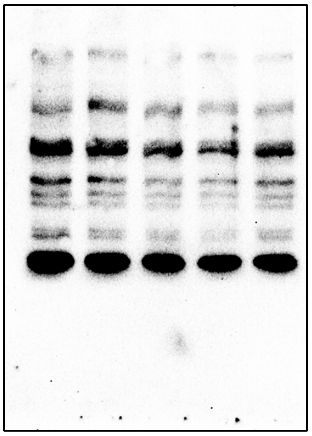

Cyclooxygenase-2

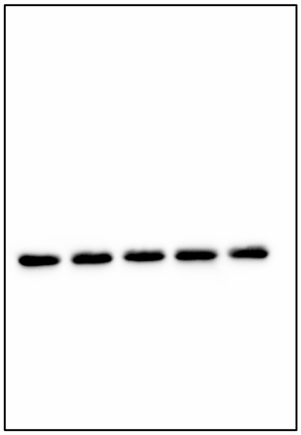

Beta actin

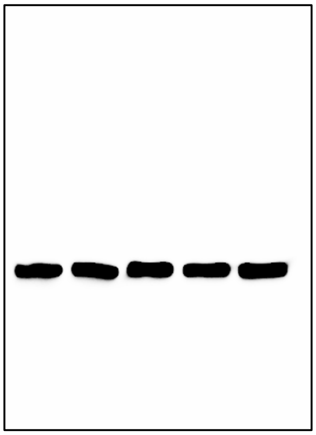

Beta actin

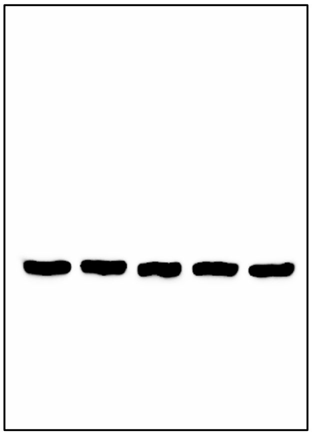

Beta actin
